# RGMa Participates in the Blood–Brain Barrier Dysfunction Through BMP/BMPR/YAP Signaling in Multiple Sclerosis

**DOI:** 10.3389/fimmu.2022.861486

**Published:** 2022-05-18

**Authors:** Lei Zhang, Shi Tang, Yue Ma, Junhang Liu, Philippe Monnier, Hang Li, Rongrong Zhang, Gang Yu, Mengjie Zhang, Yongmei Li, Jinzhou Feng, Xinyue Qin

**Affiliations:** ^1^ Department of Neurology, The First Affiliated Hospital of Chongqing Medical University, Chongqing, China; ^2^ Donald K. Johnson Eye Institute, Krembil Research Institute, University Health Network, Toronto, ON, Canada; ^3^ Department of Ophthalmology and Vision Science, Faculty of Medicine, University of Toronto, Toronto, ON, Canada; ^4^ Department of Physiology, Faculty of Medicine, University of Toronto, Toronto, ON, Canada

**Keywords:** multiple sclerosis, blood–brain barrier, repulsive guidance molecule-a, bone morphogenetic protein, yes-associated protein

## Abstract

The infiltration of inflammatory cells into the central nervous system (CNS) through the dysfunctional blood–brain barrier (BBB) was critical in the early stages of MS. However, the mechanisms underlying BBB dysfunction remain unknown. Repulsive guidance molecule-a (RGMa) is involved in the pathogenesis of multiple sclerosis (MS), but its role needs to be further explored. This study aimed to evaluate whether RMGa regulates BBB permeability in endothelial cells and MS, and if so, what mechanism may be involved. We created an experimental autoimmune encephalomyelitis (EAE) model in C57BL/6 mice and a human brain microvascular endothelial cell (HBMEC) culture. The permeability of the BBB is measured in response to various interventions. Our results showed that RGMa is expressed in the endothelial cells in HBMECs and EAE mice. RGMa and its signaling counterpart, bone morphogenetic protein 2 (BMP2)/bone morphogenetic protein receptor type II (BMPRII), were gradually increased as the disease progressed. Moreover, as EAE progressed and the BBB was disrupted, the downstream effector, yes-associated protein (YAP), as well as the tight junctional proteins zonula occludens 1 (ZO-1) and claudin-5, decreased significantly. The permeability assay revealed that lentivirus-induced RGMa overexpression in HBMECs caused a significant breakdown of the BBB, whereas RGMa knockdown significantly strengthens the integrity of the BBB. Furthermore, specifically activating BMPR II or inhibiting YAP based on RGMa knockdown results in a significant decrease of ZO-1 and claudin-5 *in vitro*. On the contrary, inhibition of BMPR II or activation of YAP after upregulating RGMa prevents the downregulation of ZO-1 and claudin-5 in HBMECs. In addition, serum-soluble RGMa (sRGMa) levels were significantly higher in MS patients, particularly in MS patients with Gd^+^ lesions, indicating that the BBB has been disrupted. In conclusion, this study shows that RGMa causes BBB dysfunction in endothelial cells *via* BMP2/BMPR II/YAP, resulting in BBB integrity disruption in MS and that it could be a novel therapeutic target for BBB permeability in MS.

## Introduction

Multiple sclerosis (MS) is the most common autoimmune demyelinating disease affecting the young population’s central nervous system (CNS) (1). The clinical manifestations range from nonspecific fatigue to specific neurological dysfunctions (2). Although the pathogenesis of MS remains elusive, infiltration of inflammatory cells into the CNS as a result of blood–brain barrier (BBB) disruption is the first sign of CNS lesion formation (3). T and B lymphocytes, as well as macrophages, attack CNS parenchyma, resulting in clinical function deterioration following transendothelial migration through a disrupted BBB (4). However, the pathogenesis underlying the BBB disruption remains unclear.

RGMa is a GPI-anchored membrane protein that exerts functions on axon guidance and axonal regeneration (5), which is involved in CNS diseases including ischemic cerebral infarction (6), multiple sclerosis (7, 8), epilepsy (9), Parkinson’s disease (10), and spinal cord injury (11). Studies have found that the expression of RGMa has significantly increased in experiment autoimmune encephalomyelitis (EAE) mice. The invasion of inflammatory factors such as INF-γ, IL-17, and IL-2 into the CNS was significantly reduced after treatment of RGMa-neutralizing antibody, with significant improvement in the demyelination and neurological function (7). The underlying mechanism, however, remains unknown. This finding prompted us to explore how RGMa could regulate lymphocytes into CNS *via* the BBB.

RGMa is a coreceptor for bone morphogenetic protein (BMP) (12). However, it is unclear what biological role RGMa plays on the BBB *via* BMP/BMPR signaling. Yes-associated protein (YAP) is one of the main effectors of the Hippo signaling pathway and participates in transcriptional regulation as a transcriptional coactivator (13). According to the literature, YAP is involved in the regulation of BBB stability (14, 15). Besides that, phosphorylation of Smad was mediated by the recruitment of YAP to the phosphorylated linker sites (16). Also, Smad1/4 as BMP2 downstream effectors had a competition with YAP, interacting with TAED1 and inhibiting the cotranscriptional activity of YAP (17). All of these findings raise the following questions: (1) whether RGMa mediates BBB dysfunction, (2) what function does BMP/YAP play in BBB dysfunction, and (3) whether RGMa influences YAP in MS *via* BMP/BMPR signaling.

This study aims to explore whether RGMa mediates the dysfunction of BBB integrity and the potential signal pathway involved.

## Methods

### Experimental Animals

C57BL/6 mice (18–22 g, 6–8 weeks) were supplied by the Laboratory Animal Center of Chongqing Medical University (Chongqing, China). All mice were maintained on a 12-h light and dark cycle with *ad libitum* access to water and food. All animal operations were approved by the Ethics Committee of the First Affiliated Hospital of Chongqing Medical University.

### Preparation of Model Reagents and EAE Model

Complete Freund’s adjuvant (CFA) contained 100 mg of M. Tuberculosis H37 Ra (Mtb, 231141, BD, USA) powder in 10 ml of incomplete Freund’s adjuvant (FIA, F5881, Sigma, USA) at a final concentration of 10 mg/ml. MOG35-55 peptide powder (051716, GL Biochem, Shanghai) was dissolved in a 0.9% saline solution to a final concentration of 3.5 mg/ml, and a 1:1 ratio of MOG35-55 peptide solution and CFA/Mtb was mixed, yielding a final concentration of MOG35-55 of 1.75 mg/ml in the antigen-CFA/Mtb emulsion. Pertussis toxin (PTX, 81236A1, List Biological Lab, USA) was dissolved in a 0.9% saline solution to form a 4-µg/ml solution.

The EAE model was performed as described previously (18). Briefly, C57BL/6 mice were weighed and anesthetized with 0.8% sodium pentobarbital at 10 ml/kg. Subcutaneously, 200 µl of antigen-CFA/Mtb emulsion was injected on both sides of the spine, followed by an intraperitoneal injection of PTX. A second dose of PTX was administered on day 2 postimmunization.

### Behavioral Assessment and Neural Deficit Score

Mice were weighed and clinical scores were assessed daily by the same investigator for 21 days postimmunization. The clinical score of EAE mice was graded blindly and divided into 5 levels as follows: 1, tail moribund; 2, tail paralysis with unilateral limb weakness; 3, bilateral limb paralysis; 4, four-limb paralysis; and 5, moribund or death (7, 19).

### Permeability Measurement by Evans Blue Extraction

EAE mice were enrolled on days 7, 14, and 21 postimmunization. EAE and control group mice were tail vein injected (IOCV) for 3 ml/kg with 2% Evans Blue dye (EBD) (E-2129, Sigma-Aldrich, USA) (20). Mice were anesthetized and transcardially perfused with cold saline 2 h postinjection. Brain and spinal cord tissue samples were collected. Tissue homogenization was accomplished using 50% trichloroacetic (TCA, T818878, MACKLIN, Shanghai) acid. Homogenate was centrifuged at 16,000×*g* for 20 min, and the supernatant was diluted fourfold with ethanol. The absorbance of the EBD solution was measured at 620 nm by the multimode Plate Reader (Perkin Elmer, 139959, Singapore).

### Cell Culture and Passage

Human brain microvascular endothelial cells (HBMECs) were purchased from Pricells Company (HUM-CELL-0101, Wuhan). Passages 2 to 8 of HBMECs were used. Cells were plated into 75 cm^2^ cell culture flasks and cultured in Dulbecco’s modified Eagle’s medium: Nutrient Mixture F-12 (DMEM/F-12; C11330500BT Gibco, Waltham, MA, USA) supplemented with 10% FBS (S-FBS, Scitecher, Beijing) and 1% primary vascular endothelial cell growth supplement (Pricells, SUP-0002). Cells were incubated in 5% CO_2_ at 37°C incubators. Cells were passaged when their density reached 90%.

### siRNA Transfection, Lentivirus, and Drug Intervention

#### siRNA Transfection

Cells reaching a density of 70%–80% were used. siRNAs were purchased from GenePharma. siRNA-RGMa sense: 5′-GCCUGAAGAUCACUGAGAATT-3′, antisense: 5′-UUCUCAGUGAUCUUCAGGCTT-3′; siRNA-BMPR II: sense: 5′-GCCGAACUAAUUCCAAUAATT-3′; 5′-GUCCACCUCAAUUCAUUUAATT-3′; 5′-GGGACAUAAAUCUUGUAAATT-3′; antisense: 5′-UUAUUGGAAUUAGUUCGGCTT-3′; 5′-UUAAAUGAAUGAGGUGGACTT-3′; 5′-UUUACAAGAUUUAUGUCCCTT-3′. siNC: sense: 5′-UUCUCCGAACGUGUCACGUTT-3′; antisense: 5′-ACGUGACACGUUCGGAGAATT-3′. siRNAs were incubated with lipofectamine 2000 (Invitrogen) for 20 min. The siRNA-lipofectamine 2000 mixtures were then added to the cells with new culture media. Cells were handled 48 h postinduction.

#### Lentivirus Intervention

Cells were calculated and seeded onto the plates. Human LV-RGMa (NM-001166283.1) and negative control LV-NC were purchased from GenePharma. LV-RGMa was added into the cell culture media with a MOI of 40. Culture media were replaced after 3 days, and cells were handled the other day.

#### Drug Intervention

Drugs were included in mnTBAP chloride, a specific activator of BMPR II, verteporfin (VP), a specific inhibitor of YAP, and 1-oleoyl lysophosphatidic acid (LPA), a specific activator of YAP. Cells were incubated with 100 µM mnTBAP (HY-126397, MCE, USA) for 1 h (21), and 2 µM VP (HY-B0146, MCE, USA) for 24 h (22), and 20 µM LPA (MED23196, Medbio, Shanghai) for 2 h (23, 24).

### *In Vitro* Permeability Assay

Matrigel matrix was diluted with cold 0.01 M (pH 8.0) Tris with 0.7% NaCl. Transwell inserts were coated with the coating buffer, and they were incubated at 37°C for 2 h. Cell suspension was plated on the upper insert with 1*10^5^ cells, and the cells were transfected with LV-RGMa or LV-NC the following day. Four days later, 10 µg/ml of 4 or 70 kDa FITC-dextran was added into the upper chambers for 90 min. Inserts were then removed, and the fluorescence signal in the lower chamber media was detected (485/535) by the multimode Plate Reader (Perkin Elmer, 139959, Singapore). The absolute permeability coefficient, *P* (cm/s) was calculated using the following equation:


$$P=\left[Ct-C\left(t_{0}\right)\right]*V/A*C_{0}*t$$

where *C*(*t*) is the concentration (μg/ml) of FITC-dextran in the samples taken from the receiver wells after 90 min. *C*(*t*_0_) is the FITC-dextran concentration (μg/ml) of the samples taken after 0 min. *C*_0_ is the initial concentration (μg/ml) of the upper chamber. *t* is the duration of the flux (s). *V* is the volume (cm^3^) in the lower chamber. *A* is the surface of the transwell membrane (cm^2^). Finally, a 0.05% crystal violet staining solution was used to study the structural integrity of the HBMECs. Images were captured using an orthographic microscope (Leica DM500, Germany)

### Sample Preparation and Western Blot

#### Sample Preparation

Tissues and cells were lysed in radio immunoprecipitation buffer (P0013B Beyotime, China) with a protease inhibitor cocktail (Hy-K0010, MCE) and cleared of debris by centrifugation at 16,000×*g* for 20 min at 4°C. The concentration of proteins was measured by bicinchoninic acid (BCA) protein assay reagent (P0010Sl Beyotime, China). The lysates were boiled with SDS-loading buffer (P0286, Beyotime) at 95°C for 10 min. All protein samples were stored at −80°C until needed.

#### Western Blot

Electrophoresis gel, electrophoresis solution, electrophoresis solution, and tris-buffered saline with 0.1% Tween (TBST) were prepared prior. BCA was used to determine protein concentration, and each well was loaded equally. The gels were transferred to a 0.45-µm PVDF membrane (A10178785, GE, USA); 5% nonfat milk in TBST was used to block the membrane for 1 h at room temperature. The membranes were then incubated at 4°C overnight with primary antibodies for 16 h. The primary antibodies were as follows: anti-RGMa (1:10,000, ab169761, Abcam, USA); anti-BMP2 (1:1,000, YT5651, Immunoway, USA); anti-BMPR II (1:500, 220550, ZEN Bio, Chengdu); anti-YAP (1:1,000, 14074, Cell Signaling Technology, USA); anti-ZO-1(1:1,000, 40-2200, ThermoFisher Scientific, USA); anti-GAPDH (1:10,000, 20494-1-AP, Proteintech, Wuhan); and anti-beta Tubulin (1:4,000; 10068-1-AP, Proteintech, Wuhan). Membranes were then incubated with horseradish peroxidase-conjugated secondary antibodies (goat anti-rabbit IgG, SA-00001-2, Proteintech; goat anti-mouse IgG, SA00001-1, Proteintech) for 1 h at room temperature. Images were captured by the Fusion FX5 image analysis system (Vilber Lourmat, F77601 Marne-la-Vallée cedex 3, France).

### Immunofluorescence

Mice were sacrificed under anesthesia and transcardially perfused with cold saline followed by 4% paraformaldehyde. The brain and spinal cord were carefully removed, postfixed, dehydrated, and sectioned into 30 µm slices. After antigen retrieval, slices were permeabilized using 0.1% Triton X-100 and blocked with 10% normal donkey serum for 1 h at 37°C, and then incubated with the primary antibodies at 4°C overnight. Primary antibodies were as follows: anti-CD31 (1:500, ab9498, Abcam, USA); anti-RGMa (1:50, ab179761, Abcam, USA); anti-BMP2 (1:100, 18933-1-AP, Proteintech, Wuhan); anti-BMPR II (1:200, AF5385, Affinity, Jiangsu); anti-YAP (1:100, 14074, Cell Signaling Technology, USA). After washing with PBS, the slices were incubated with the secondary antibodies for 1 h at room temperature. For HBMECs, cells were fixed with 4% sucrose paraformaldehyde. After permeabilizing with 0.1% Triton X-100 and blocking with 10% normal donkey serum for 1 h at 37°C, specific primary antibodies were incubated at 4°C overnight. Secondary antibodies (594, goat anti-mouse IgG, A23410, Abbkine, 1:200, USA; 488, goat anti-mouse IgG; A23220, Abbkine, 1:200) were incubated for 1 h at room temperature after washing with PBS. Images were captured by the Nikon system (Nikon ECLIPSE Ci-E, OFN25 DIC, Japan).

### Human Blood Sample Collection

All serum samples of MS patients were collected from December 2017 to June 2021, in the Department of Neurology at the First Affiliated Hospital of Chongqing Medical University. The diagnoses of all the patients were reviewed, and only the patients who fulfilled the McDonald 2017 criteria were included (25). The following patients were excluded: (1) those with malignant tumors; (2) those with the other inflammatory demyelination disease; (3) those with severe hepatic or renal insufficiency; (4) those who did not have an RGMa laboratory result; and (5) those who received acute therapy within 7 days. The protocol was approved by the Ethics Committee of the First Affiliated Hospital of Chongqing Medical University and adhered to the ethical principles for medical research involving human subjects of the Helsinki Declaration. All the patients signed an informed consent. Blood samples were allowed to coagulate at 4°C overnight, followed by centrifugation at 2,000×*g* for 10 min. Serum was then collected and stored at −80°C for usage.

### Enzyme-Linked Immunosorbent Assay

Serum-soluble RGMa (sRGMa) expression was detected by a commercial RGMa enzyme-linked immunosorbent assay (ELISA) kit (DY2459-05, R&D, USA). The protocols were carried out in accordance with the manufacturer’s instructions. Briefly, the process was as follows: the 96-well plates were coated with capture antibody in PBS overnight. The plates were blocked with reagent diluent concentrates for 1 h. Subsequently, the standard protein and diluent serum samples were added to each well and incubated for 2 h. The detection antibody was then added to the reagent diluent and incubated for another 2 h. Following that, streptavidin-HRP was added to the plate for 20 min while avoiding direct light. The plates were then washed 3–5 times with wash buffer concentrates after each solution was incubated. After the streptavidin-HRP was removed and thoroughly washed, the TMB substrate (BU04, NEOBISCOENCE, Shanghai) was added to the well and incubated for another 20 min while avoiding direct light. Finally, a stop solution (C1058, Solarbio, Beijing) was used to stop action. All operations were performed at room temperature. The multimode Plate Reader (Perkin Elmer, 139959, Singapore) detected absorbance at 450 nm and calibrated at 540 nm.

### Assessment of BBB Function in MS Patients

Lesions with gadolinium enhancement (Gd^+^) on magnetic resonance imaging (MRI) and Qalb were adopted as indicators of BBB disruption (4). The cerebrospinal fluid (CSF)/serum albumin quotient, QAlb = AlbCSF (mg/L)/Albserum (g/L), was used to assess the BBB function in MS patients. According to a previous study, the upper reference limit of QAlb is age dependent, Qlim(Alb) was calculated as 4 + (*a*/15) × 10^−3^ with *a* representing patient’s age (26). Dysfunction of the BBB was defined as QAlb > Qlim(Alb).

### Statistical Analyses

All the data presented represent results from at least three independent experiments. All statistical analyses were conducted by SPSSC22.0; graphs were generated by Graphpad Prism 6. The mean ± standard error of the mean (SEM) was used to express quantitative data, the Shapiro–Wilk test was used to test for normality test, *t*-tests were used for independent comparisons, and ANOVA and post-Bonferroni with *post-hoc* tests were used to compare multiple groups. Enumeration data were compared by Chi-square test. *p*-values less than 0.05 were considered significant.

## Results

### RGMa Mediates BBB Breakdown in EAE Mice

**Figure 1:**
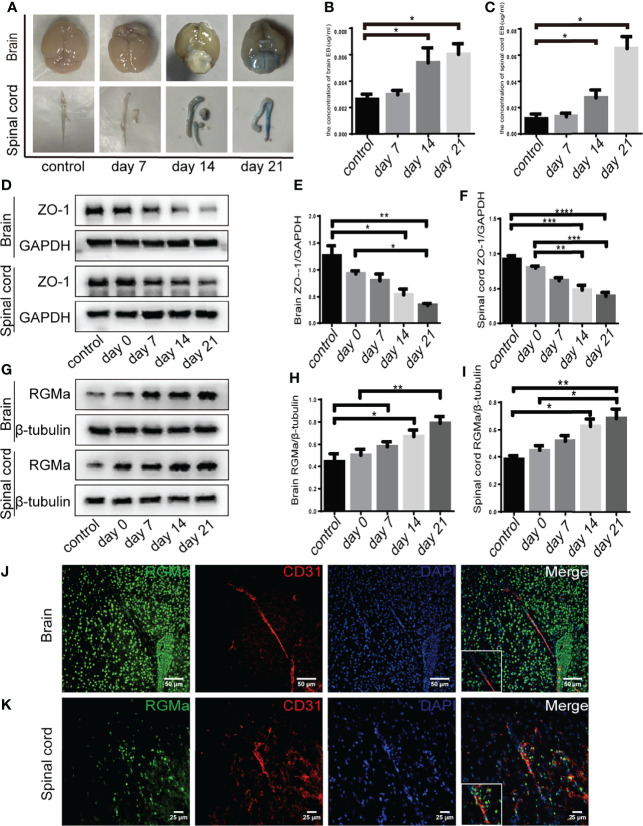
RGMa is associated with blood–brain barrier disruption in EAE mice. **(A–C)** Quantitative analysis of Evans Blue Dye staining showed BBB disruption was significantly higher in both the 14-day group and the 21-day group compared with the control group (control vs. 14 days: brain, ^*^ *p* < 0.05; spinal cord, ^****^ *p* < 0.0005; control vs. day 21: brain, ^*^ *p* < 0.05; spinal cord, ^*^ *p* < 0.05). **(D–F)** In both the brain and spinal cord, ZO-1 expression levels were decreased at both 14 and 21 days compared with the control group or 0-day group. **(G–I)** RGMa expression was significantly increased in the brain and spinal cord at 14 and 21 days compared with the control or 0-day groups, and significantly increased at the 21-day group compared with 7-day group (*n* = 4, error bar: SEM; ^*^ *p* < 0.05, ^**^ *p* < 0.01, ^***^ *p* < 0.001, ^****^ *p* < 0.0001, one-way ANOVA with Bonferroni). **(J–K)** RGMa and CD31^+^ endothelial cells were found colocalized in the brain cortex and spinal cord white matter. Scale bar: 50 µm (brain), 25 µm (spinal cord).

EBD extravasation was detected in the BBB permeability of the EAE mouse model. The concentration of EBD was slightly increased in the 7-day group compared with the control group (*p* > 0.05; **Figures 1A** **–C**). The accumulation and concentration of EBD were significantly higher in both the 14-day group and the 21-day group compared with the control group (*p* < 0.05; **Figures 1A** **–C**). Meanwhile, Western blot showed that both brain and spinal cord ZO-1 expressions were significantly decreased in the 14-day group (*p* < 0.05) and 21-day group (*p* < 0.01) compared with the control group (**Figures 1D** **–F**). These results verified severe BBB disruption in EAE mice.

Our previous research demonstrated that RGMa is involved in the BBB dysfunction in a rat model of middle cerebral artery occlusion/reperfusion (27). To investigate the effect of RGMa on BBB permeability in the EAE model, we first measured RGMa expression in the brain and spinal cord by Western blot. RGMa expression levels in both the brain and spinal cord were significantly elevated in the 14-day group (p < 0.05) and the 21-day group (p < 0.01) compared with the control group **(** **Figures 1G** **–I** **)**. Meanwhile, immunofluorescence showed strong staining of RGMa colocalizing with CD31+ endothelial cells in the brain and spinal cord sections of EAE **(** **Figures 1J** **–K** **).**

### RGMa Mediates Integrity of BBB *In Vitro*

**Figure 2:**
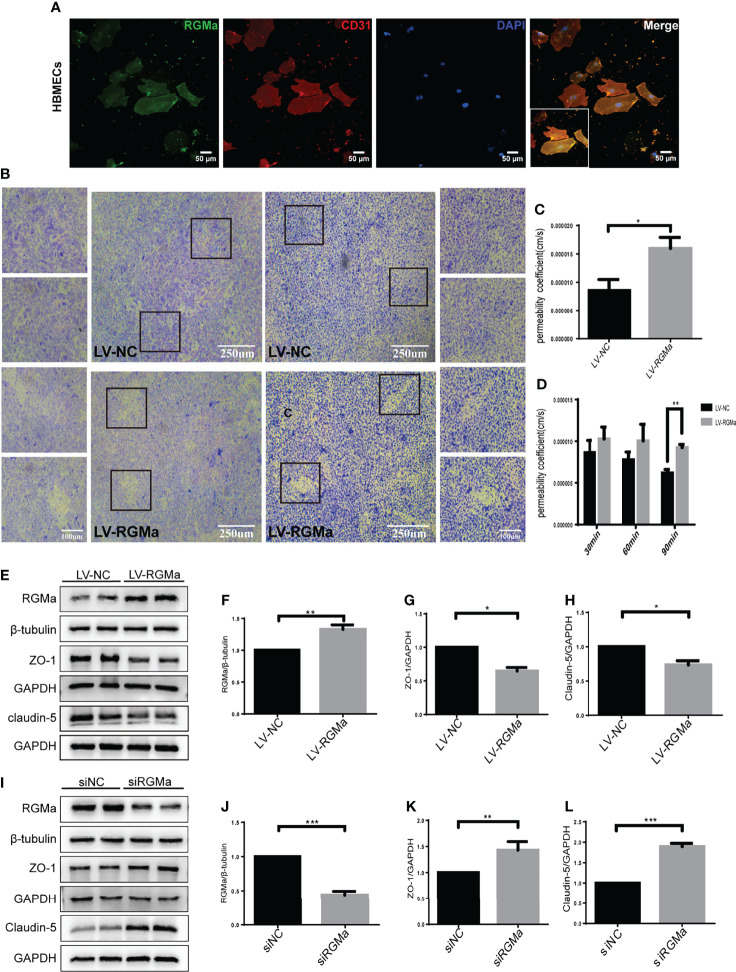
RGMa deteriorates the integrity of the *in vitro* BBB by HBMECs. **(A)** RGMa was expressed in CD31^+^ HBMECs. Scale bar: 50 µm. **(B)** The permeability of *in vitro* BBB was detected by crystal violet staining and FITC-dextran-4 kDa (right) or 70 kDa (left) assay. Overexpression of RGMa significantly exacerbates BBB permeability (error bar: SEM; ^**^ *p* < 0.01, scale bar: 250 µm). **(C)** After 90 min, the permeability coefficient of FITC-dextran-70 kDa in the LV-NC and LV-RGMa groups. **(D)** After 90 min, the permeability coefficient of FITC-dextran-4 kDa in the LV-NC and LV-RGMa groups. **(E–L)** By specifically upregulating or downregulating RGMa, the expression of ZO-1 and claudin-5 were accordingly decreased or increased (*n* = 3, error bar: SEM; ^*^ *p* < 0.05, ^**^ *p* < 0.01, ^***^ *p* < 0.001, *t*-test).

To confirm the role of RGMa in the dysfunction of the BBB, we cultured the HBMECs as an *in vitro* BBB model. Immunofluorescence results verified that RGMa was expressed in CD31^+^ HBMECs (**Figure 2A**). Besides, the Western blot showed that RGMa was overexpressed in the LV-RGMa group compared with the LV-NC group (*p* < 0.05). The results of the monolayer transwell permeability assay with FITC-dextran-4 kD or FITC-dextran-70 kDa (permeability to small or larger molecules) and crystal violet staining illustrated a significant disruption of HBMEC permeability upon upregulating RGMa with LV-RGMa (*p* < 0.01; **Figures 2B** **–D**). In addition, ZO-1 and claudin-5 expression levels were significantly decreased in the LV-RGMa group (*p* < 0.05; **Figures 2E** **–H**). On the contrary, after RGMa was specifically knocked-down in the siRGMa group (*p* < 0.001), the expressions of ZO-1 and claudin-5 were significantly increased compared with the siNC group (*p* < 0.01; **Figures 2I** **–L**). Taken together, these results suggested that RGMa mediates the disruption of the BBB in HBMECs.

### Intervention of BMPR II Altered the Integrity of BBB Induced by RGMa

**Figure 3:**
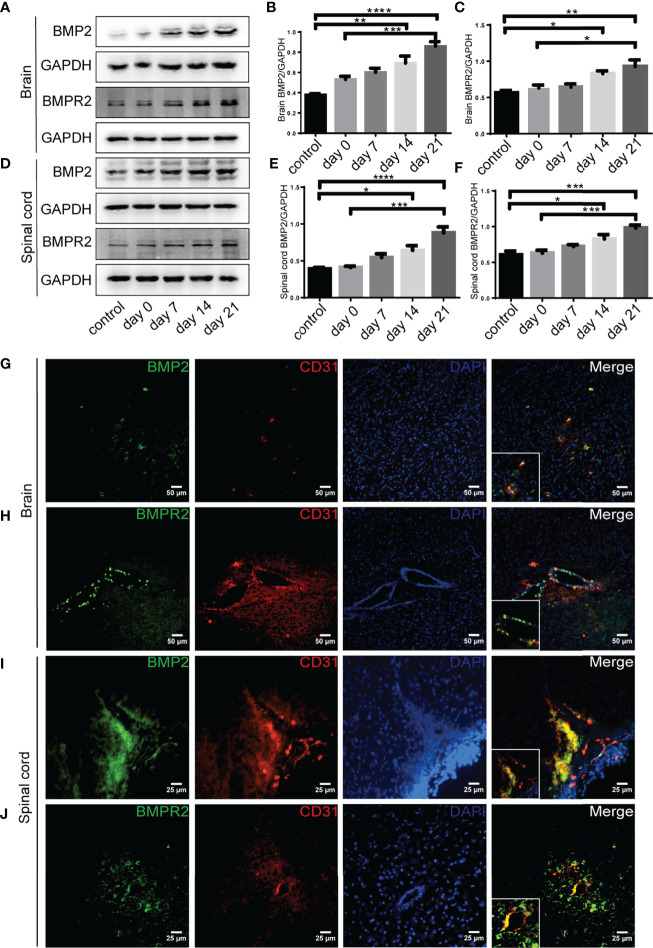
BMP2 and BMPR II expression in EAE mice. **(A–F)** BMP2 and BMPR II in the brain and spinal cord were both upregulated in the 14- and 21-day group compared with the control group. BMP2 and BMPR II in both the brain and spinal cord were significantly higher in the 21-day than the 0-day groups (*n* = 4, error bar: SEM; ^*^ *p* < 0.05, ^**^ *p* < 0.01, ^***^ *p* < 0.001, ^****^ *p* < 0.0001, one-way ANOVA with Bonferroni). **(G–J)** Immunofluorescence showed BMP2 and BMPR II were located in CD31^+^ endothelial cells in both the brain cortex and the white matter of the spinal cord. Scale bar: 50 µm (brain), 25 µm (spinal cord).

**Figure 4:**
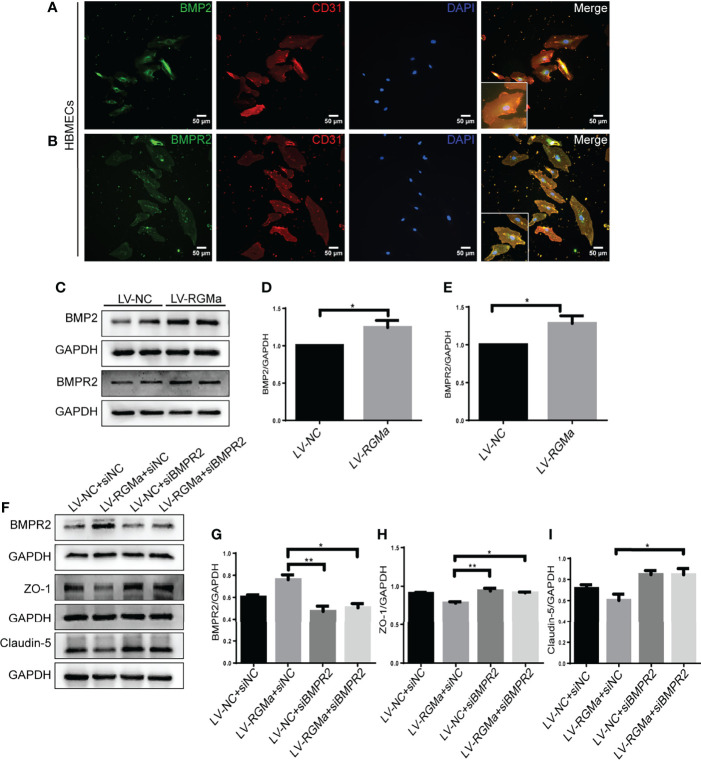
BMPR II mediates the integrity of the BBB induced by RGMa. **(A, B)** Immunofluorescence showed BMP2 and BMPR II were expressed in HBMECs. Scale bar: 50 µm. **(C–E)** Both BMP2 and BMPR II expression levels were significantly increased after overexpressing RGMa (*n* = 3, ^*^ *p* < 0.05, *t*-test). **(F–I)** On the basis of overexpressing RGMa, specifically silencing BMPR II significantly augments ZO-1 and claudin-5 expression (*n* = 3, ^*^ *p* < 0.05, *t*-test). **p < 0.01.

**Figure 5:**
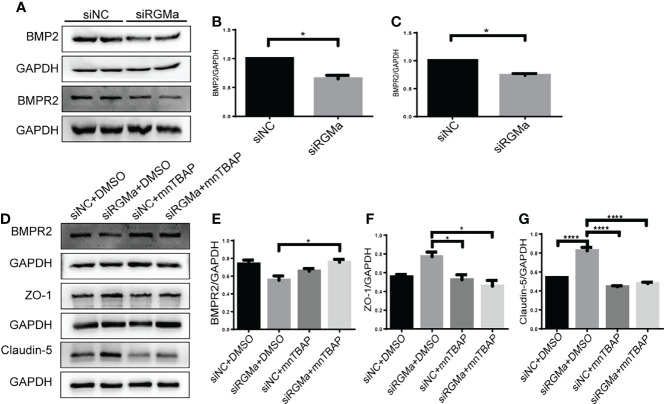
RGMa regulates BMP2 and thereby tight junctional protein expression. **(A–C)** BMP2 and BMPR II expression were significantly decreased after RGMa knockdown *in vitro* BBB (*n* = 3, ^*^ *p* < 0.05, *t*-test). **(D–G)** After activating BMPR II by mnTBAP 100 µM for 1 h based on inhibiting RGMa, ZO-1 and claudin-5 expression levels were significantly reduced (*n* = 3, ^*^ *p* < 0.05, *t*-test). ****p < 0.0001.

Western blot showed that BMP2 and BMPR II expression in both the brain and spinal cord were elevated in the 14-day group (*p* < 0.05) and the 21-day group (*p* < 0.01) compared with the control group (**Figures 3A–F**). In particular, immunofluorescence showed BMP2 and BMPR II were both expressed in CD31^+^ CNS endothelial cells both *in vivo* and in HBMECs (**Figures 3G–J**, **4A, B**). Meanwhile, we discovered that both BMP2 and BMPR II expressions were significantly increased in the LV-RGMa group compared with the LV-NC group (*p* < 0.05; **Figures 4C** **–E**). To determine whether RGMa mediates BBB through BMP2/BMPR II signaling, we inhibited BMPR II by siBMPR II followed by upregulating RGMa *in vitro*. The cells were divided into four groups: the LV-NC+siNC group, the LV-RGMa+siNC group, the LV-NC+siBMPR II group, and the LV-RGMa+siBMPR II group. Western blot showed that BMPR II expression was significantly suppressed after knocking down BMPR II following the upregulation of RGMa. However, ZO-1 and claudin-5 levels were more ameliorated in the LV-RGMa+siBMPR II group than in the LV-RGMa+siNC group (*p* < 0.05; **Figures 4F** **–I**). On the other hand, BMP2 and BMPR II expression levels were significantly decreased in the siRGMa group compared with the siNC group (*p* < 0.05; **Figures 5A** **–C**). Furthermore, cells were separated into four groups: the siNC+DMSO group, the siRGMa+DMSO group, the siNC+mnTBAP group, and the siRGMa+mnTBAP group. Western blot demonstrated BMPR II was increased in the siRGMa+mnTBAP group compared with the siRGMa+DMSO group, while ZO-1 and claudin-5 were significantly decreased in si-RGMa+mnTBAP group (*p* < 0.05; **Figures 5D** **–G**). These results demonstrate that BMP2/BMPR II signaling mediates the destruction of BBB integrity induced by RGMa.

### RGMa/BMP2/BMPRII Pathway Mediates the Integrity of BBB *via* Downregulating YAP

**Figure 6:**
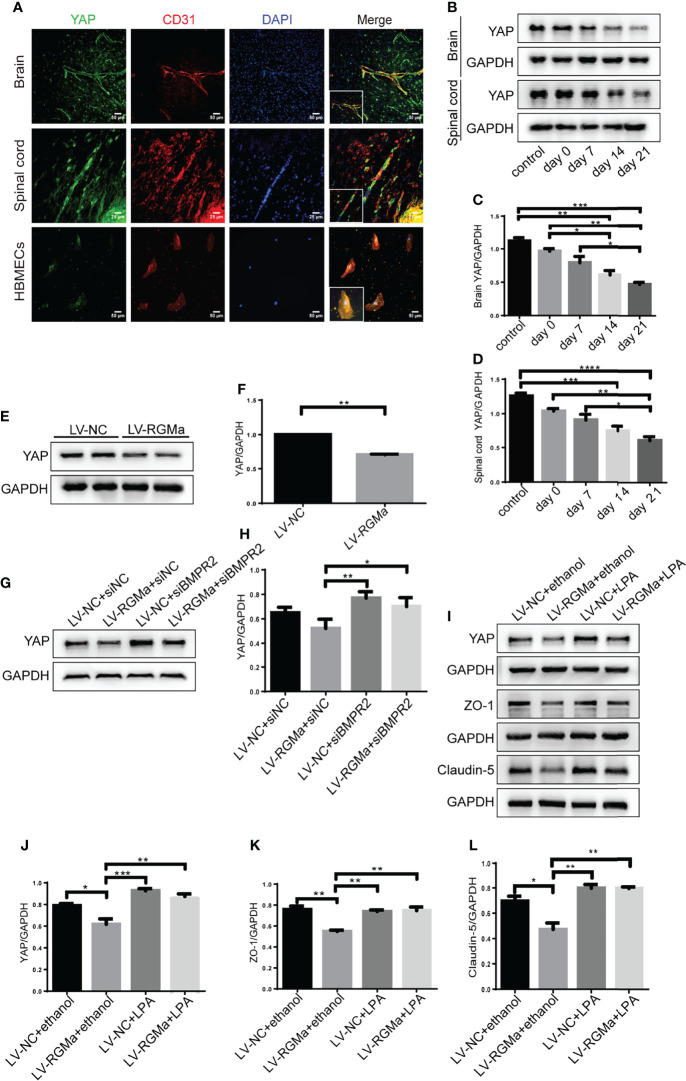
YAP regulates RGMa-induced integrity of the BBB. **(A)** Immunofluorescence showed that YAP was expressed in CD31^+^ endothelial cell both in EAE mice and HBMECs. Scale bar: 50 µm (brain cortex and HBMECs) and 25 µm (spinal cord). **(B–D)** YAP expression gradually decreased with the progression of EAE. (*n* = 4, error bar: SEM; ^*^ *p* < 0.05, ^**^ *p* < 0.01, ^***^ *p* < 0.001, ^****^ *p* < 0.0001, one-way ANOVA with Bonferroni). **(E, F)** YAP expression was significantly diminished when overexpressing RGMa *in vitro* (*n* = 3, ^**^ *p* < 0.01, *t*-test). **(G, H)** The YAP expression level was ameliorated when blocking BMPR II (*n* = 3, ^*^ *p* < 0.05, *t*-test). **(I–K)** By overexpressing YAP by LPA 20 µM for 2 h after overexpressing RGMa, both ZO-1 and claudin-5 were significantly increased (*n* = 3, ^**^ *p* < 0.01, *t*-test).

**Figure 7:**
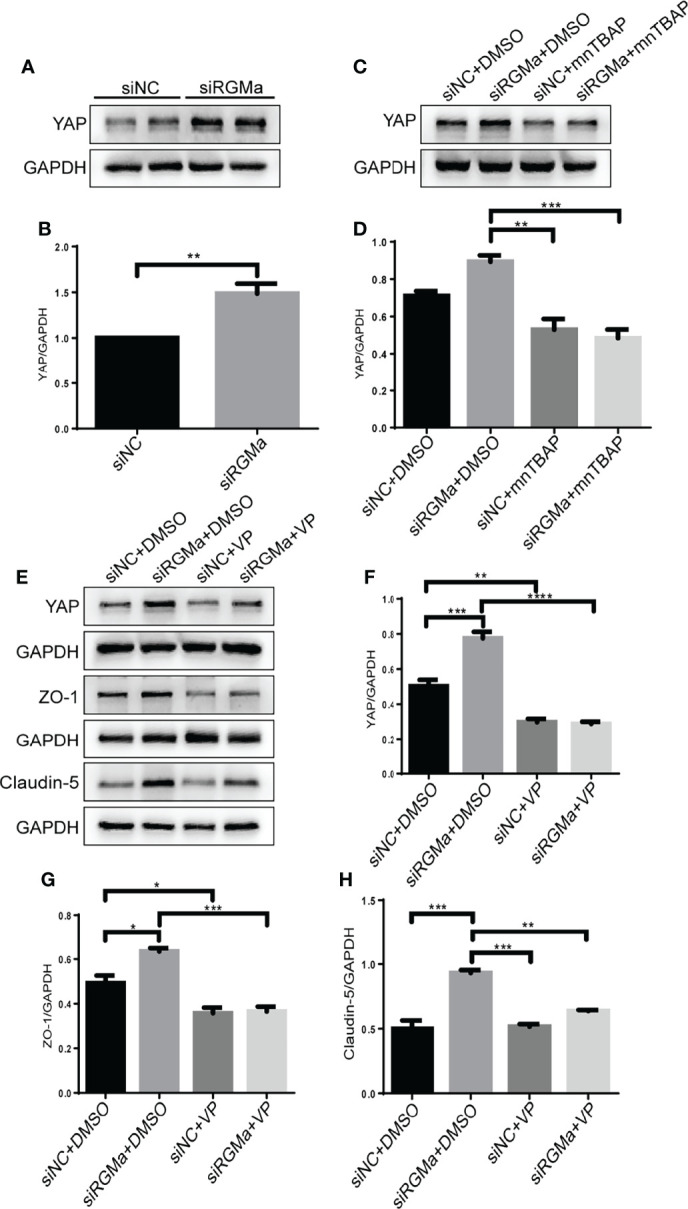
Inhibiting YAP augments the permeability of the BBB and mediates *via* RGMa/BMP pathway. **(A–D)** Western blot analysis showed that YAP was significantly upregulated in the siRGMa group (*n* = 3, **p < 0.01, *t*-test) but significantly decreased when BMPR II was activated for 1 h with mnTBAP 100 µM (*n* = 3, ***p < 0.001). **(E–H)** YAP, ZO-1, and claudin-5 levels were significantly reduced when YAP expression was specifically inhibited by VP 2 µM for 24 h on the basis of RGMa knockdown (*n* = 3, *p < 0.05, ^**^ *p* < 0.01, ^***^ *p* < 0.001, ^****^ *p* < 0.0001, *t*-test).

YAP is expressed in CD31^+^ endothelial cells both in the brain and spinal cord of EAE mice and in HBMECs *in vitro via* immunofluorescence (**Figure 6A**). YAP expression was significantly decreased in the 14-day group (*p* < 0.001) and the 21-day group (*p* < 0.0001) in the brain and spinal cord in EAE mice (**Figures 6B** **–D**). Western blot showed that the YAP expression level was significantly declined in the LV-RGMa group compared with the LV-NC group (*p* < 0.01; **Figures 6E, F** **)**. Besides that, the decrease of YAP expression was inhibited when blocking BMPR II following overexpressing RGMa (*p* < 0.05; **Figures 6G, H** **)**, illustrating that YAP, ZO-1, and claudin-5 expression levels are regulated by BMPR II *in vitro*. Next, *in vitro* groups were divided into four groups: LV-NC+ethanol group, LV-RGMa+ethanol group, LV-NC+LPA group, LV-RGMa+LPA group. By activating YAP after overexpressing RGMa *in vitro*, YAP, together with ZO-1 and claudin-5 expression levels in the LV-RGMa+LPA group, was upregulated compared with the LV-RGMa+ethanol group (*p* < 0.01; **Figures 6I** **–L**). *In vitro* BBB dysfunction was mitigated by upregulating YAP upon overexpressing RGMa. However, when we knocked down RGMa, results showed that YAP expression was increased in the siRGMa group than in the siNC group (*p* < 0.01; **Figures 7A, B** **)**. Nevertheless, YAP expression level was decreased when nmTBAP was added into siRGMa+mnTBAP group, compared with siRGMa+DMSO group (*p* < 0.001; **Figures 7C, D** **)**. To assess whether YAP was affected by RGMa-BMP2/BMPR II pathway, we inhibited YAP based on knocking down RGMa. Cells were divided into four groups: siNC+DMSO group, siRGMa+DMSO group, siNC+VP group, and siRGMa+VP group. By inhibiting YAP after downregulating RGMa, Western blot manifested that YAP, ZO-1, and claudin-5 expression levels were significantly decreased in the siRGMa+VP group compared with the siRGMa+DMSO group (*p* < 0.01), consistent with the results in the siRGMa+mnTBAP group (**Figures 7E** **–H**). Taken together, RGMa mediates the integrity of BBB through BMP2/BMPR II pathway by downregulating YAP.

### Soluble RGMa Levels in Serum Is Significantly Higher in MS Patients and Associated With the Disruption of BBB

**Figure 8:**
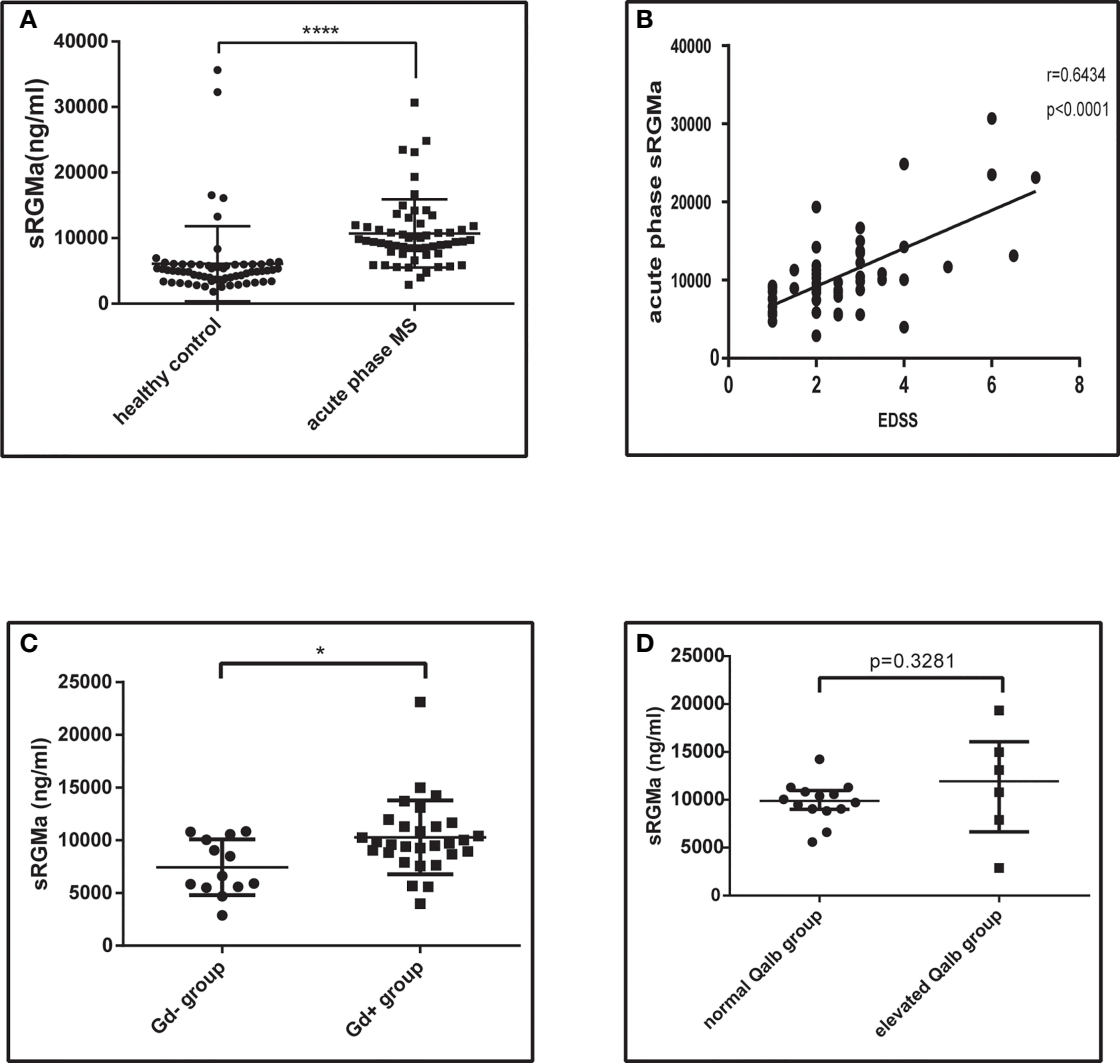
Serum RGMa expression and correlation with BBB permeability in MS patients. **(A)** ELISA detected a significantly higher level of sRGMa expression in acute phase MS patients than in the HC group (10,706 ± 689.3 ng/ml vs. 6,081 ± 723.0 ng/ml, error bar: SD; ^****^ *p* < 0.0001). **(B)** Pearson correlation coefficient showed a significant correlation between EDSS at admission and sRGMa in acute phase MS (*p* < 0.0001, *r* ^2^ = 0.6434). **(C)** ELISA detected significantly higher levels of sRGMa expression in the Gd^+^ group than in the Gd^−^ group by (7,448 ± 734.5 ng/ml vs. 10,283 ± 650.6 ng/ml, error bar: SD; ^*^ *p* < 0.05). **(D)** ELISA detected a slightly increased level of sRGMa expression in the elevated Qalb group than in the normal Qalb group (9,883 [8,592, 10,993] vs. 11,956 [5,499, 17,517] ng/ml, median, 95% CI, *p* = 0.3281, Kolmogorov–Smirnov test).

Serum samples from 57 acute phase MS patients and 63 healthy controls were obtained. Participants enrolled were age- and sex-matched (**Table 1**). We first analyzed the sRGMa expression level between acute phase MS patients and healthy controls and found that the sRGMa expression level was significantly higher in the MS acute phase than in healthy controls (**Table 1**; **Figure 8A**). Moreover, a significantly positive correlation was observed between the EDSS score at admission and the sRGMa level in MS patients (**Figure 8B**). We also found that sRGMa levels are significantly higher in MS with Gd^+^ lesions than Gd^−^ lesions (*p* < 0.05; **Figure 8C**). Besides, sRGMa levels were slightly increased in the elevated Qalb group than in the normal Qalb group without any statistical difference (*p* > 0.05; **Figure 8D**). These results demonstrated that serum sRGMa levels are associated with the disruption of the BBB in MS.

**Table 1:** Demographic features of clinical participants.

	HC (*N* = 63)	MS (*N* = 57)	*p*-value
Age (mean ± SEM)	36.65 ± 1.357	33.75 ± 1.651	0.1747
Sex (female, %)	46 (73.02%)	41 (71.93)	0.8940
sRGMa (ng/ml)	6,081 ± 723.0	10,706 ± 689.3	<0.0001

## Discussion

**Figure 9:**
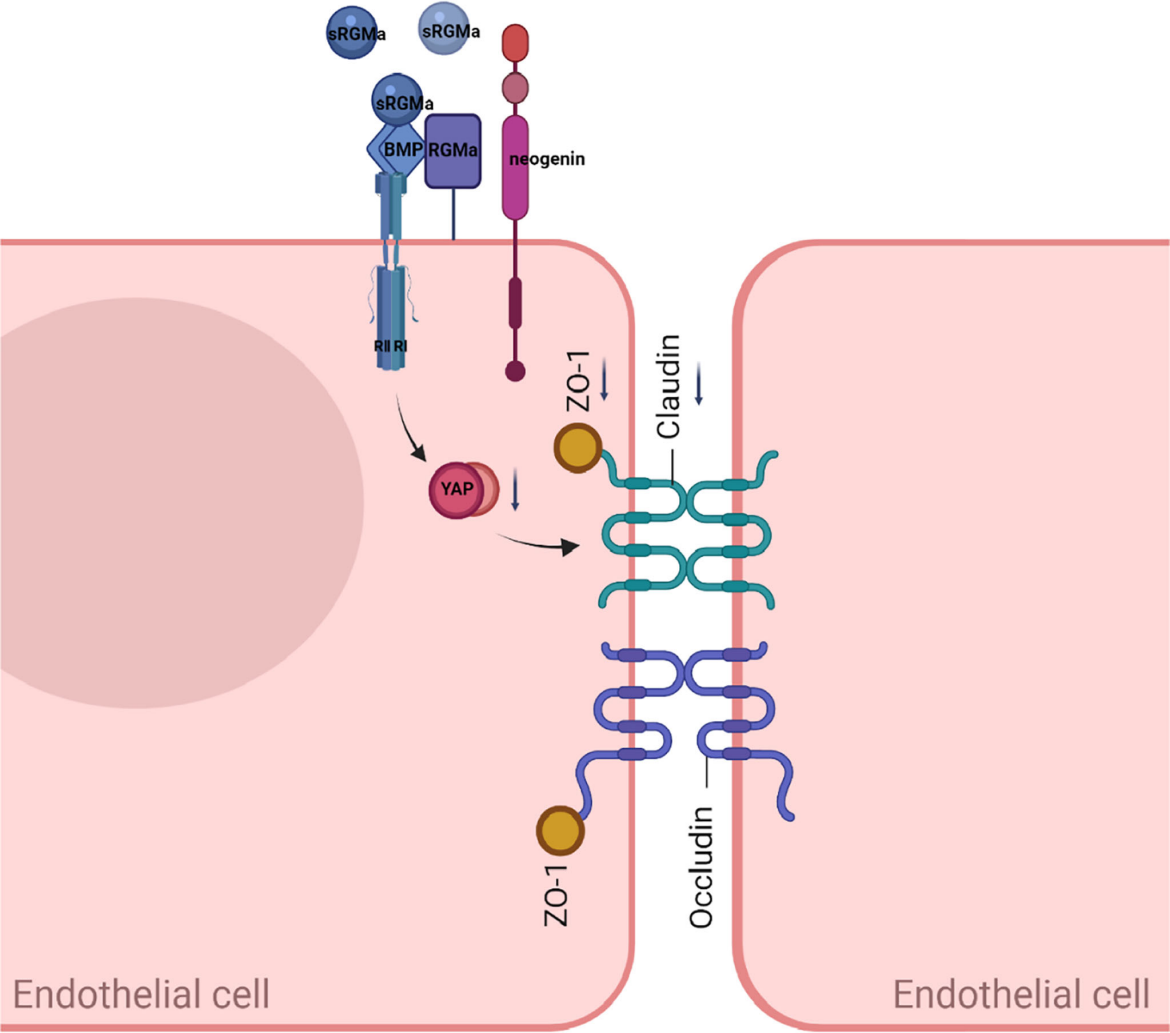
A proposed model of RGMa regulation of BMP2/BMPR II/YAP signaling in endothelial cells. The overexpression of RGMa forms a complex by binding BMP2 and BMPR II, then attenuates YAP expression, which promotes the disruption of the BBB. By regulating the BMP2/BMPR II/YAP pathway, RGMa mediates dysfunction of the blood–brain barrier.

In this study, we present evidence for the critical role of RGMa in the regulation of the integrity of BBB *in vitro* and proposed the working model shown in **Figure 9**. Overexpression of RGMa increases the level of BMP2 expression, which activates its cell surface receptor BMPR II. Forming a complex with BMPR II then inhibits the expression of YAP, which ultimately leads to the dysfunction of BBB permeability. This is the first study to investigate the roles and mechanisms of RGMa on the BBB in MS.

RGMa, which is expressed by a wide range of cells, regulates a variety of functions by relying on its cellular and environmental context (28–30). Here, we demonstrated that RGMa is expressed in endothelial cells in EAE, which is consistent with our previous study on stroke (30). The permeability of an *in vitro* BBB model is increased after overexpressing RGMa *via* lentiviral intervention. In HBMECs, knocking down RGMa has the opposited effect. Meanwhile, BBB permeability was assessed using crystal violet and FITC-dextran, and the expression of tight junctional proteins such as ZO-1 and claudin-5 was significantly altered *in vitro* following RGMa intervention. The results showed that RGMa plays a vital role in BBB disruption. Since RGMa is expressed not only in the central nervous system but also in the peripheral nervous system, it is unclear whether RGMa located in endothelial cells is cleaved by itself or secreted by peripheral immune cells to perform the BBB permeability mechanism.

Previous studies have shown that RGMa, binding either to neogenin or BMP/BMPR, exerts its function. Firstly, RGMa binds to the transmembrane protein neogenin and acts on axonal regeneration (31), tube formation (32), and cell death (33). Secondly, RGMa could function as a BMP coreceptor (12, 34). BMPs transduce their signals by binding to and stabilizing membrane complexes consisting of types I and II receptors, thereby inducing Smad1/5/8-dependent and non-Smad-dependent signaling (35, 36). However, the biological function of BMP signaling related to RGMa has not been clearly illustrated. Previous findings reveal that BMPs have a pivotal role in the endothelial cells both in central BBB and peripheral circumstances (37, 38). Based on this evidence, we focused on the role of BMP/BMPR signaling in RGMa-mediated BBB dysfunction.

Our current study shows that RGMa, BMP2, and BMPR II expression levels are progressively upregulated, accompanied by the BBB dysfunction in the EAE model. We then applied an *in vitro* HBMEC BBB model and indicated that exacerbated BBB permeability by overexpressing RGMa *in vitro*, which is consistent with the EAE model. Results also showed that specific intervention of RGMa regulates BMP2 and BMPR II expressions, thereby acting on BBB junctional proteins such as ZO-1 and claudin-5 expressions. Another study shows that RGMa causes BBB dysfunction (27, 39), but the model and hypothesis are different. As to another receptor, a previous study found that neogenin appears to be a receptor of BMPs bridged by RGMa, which strengthens or weakens BMP signaling (12). However, how neogenin modulates BMP signaling is still controversial. As a result, the roles of neogenin in the interaction act between RGMa and BMPs must be investigated further.

Several studies have proposed YAP could decrease Smad1/5/8 phosphorylation and the inhibitory effect of BMP2 downstream (17, 40). The current study demonstrates YAP expression is regulated by RGMa/BMP2/BMPR II pathway in the *in vitro* BBB model. Furthermore, the effect of RGMa on the disruption of the BBB works by modulating YAP expression. Wang et al. found that YAP was decreased in ischemia/reperfusion in the brain and observed an increased level of tight junction proteins (ZO-1 and Claudin-3) and decreased BBB permeability when a YAP agonist was applied (39), which is consistent with our findings. In the last step, gadolinium enhancement on MRI and Qalb were performed as substitute indicators to evaluate BBB breakdown in MS patients. Correlation analysis results demonstrated that the expression of serum RGMa could reflect BBB disruption in MS patients.

In summary, our findings indicate that RGMa may have a critical role in BBB dysfunction in HBMECs and MS. RGMa causes BBB dysfunction by forming a molecular complex with BMP2/BMPR II and downregulating YAP expression, thereby leading to the disruption of BBB integrity. RGMa may be a novel therapeutic target *via* BBB dysfunction in MS.

## Data Availability Statement

The original contributions presented in the study are included in the article/supplementary material. Further inquiries can be directed to the corresponding authors.

## Ethics Statement

The studies involving human participants were reviewed and approved by the Ethics Committee of the First Affiliated Hospital of Chongqing Medical University. The patients/participants provided their written informed consent to participate in this study. The animal study was reviewed and approved by the Ethics Committee of the First Affiliated Hospital of Chongqing Medical University. Written informed consent was obtained from the owners for the participation of their animals in this study. Written informed consent was obtained from the individual(s) for the publication of any potentially identifiable images or data included in this article.

## Author Contributions

LZ performed all cellular experiments, mouse experiments, as well as statistical analysis. ST, YM, HL, MZ, and GY collected the clinical data. JL and YL performed the analysis of magnetic resonance imaging data. LZ drafted the manuscript. PM and RZ did critical revision of the manuscript. XQ and JF supervised the entire study. All authors listed have made a substantial, direct, and intellectual contribution to the work and approved it for publication.

## Funding

This work was supported by the National Natural Science Foundation of China to JF (No. 81701191), Chongqing Medical Scientific Research Project (Joint project of Chongqing Health Commission and Science and Technology Bureau, No. 2020FYYX104), and National Key Clinical Specialties Construction Program of China.

## Conflict of Interest

The authors declare that the research was conducted in the absence of any commercial or financial relationships that could be construed as a potential conflict of interest.

## Publisher’s Note

All claims expressed in this article are solely those of the authors and do not necessarily represent those of their affiliated organizations, or those of the publisher, the editors and the reviewers. Any product that may be evaluated in this article, or claim that may be made by its manufacturer, is not guaranteed or endorsed by the publisher.
